# The Wales Cancer Research Centre: improving cancer care together

**DOI:** 10.3332/ecancer.2017.ed65

**Published:** 2017-04-19

**Authors:** John D Chester, Jodie R Bond, Elisabeth A Batt

**Affiliations:** Wales Cancer Research Centre, Cardiff University, University Hospital of Wales, Cardiff, CF14 4XN, UK

**Keywords:** Wales Cancer Research Centre, Wales, Welsh Government, translational pipeline, partnerships

## Abstract

The Wales Cancer Research Centre (WCRC), funded by the Welsh Government, through Health and Care Research Wales, is our latest weapon in the ongoing war against cancer. It is Wales’ contribution to a global effort to reverse the increasing trend in numbers of newly diagnosed cancers. With the increasing need for new and better cancer treatments, more research is needed, and the WCRC hopes to fulfill some of this demand. WCRC is particularly concerned with the problems of cancer patients in Wales, but also aims to help cancer patients beyond Wales by performing cancer research of world class quality.

The Centre has an all-Wales brief, with a vision to work with cancer patients and other partners to deliver cancer research excellence to help patients in Wales and beyond. Our research partners include three universities and four NHS Boards and Trusts. At every stage of our work, we involve public and patient representatives. This is based on our strong conviction that ordinary people should not just be the subjects of research, but be active participants in our research, working with us to plan, conduct and present our work.

Sadly, new cases of cancer in Wales are increasing - up 12 per cent since 2004 - just as resources for the NHS are under their greatest pressure. Right now, around 120,000 people are living with cancer in Wales, and this figure is set to nearly double by 2030. The rate of cancer diagnosis in Wales is 20% higher than in England, in part due to our more economically deprived population.

As well as having its own particular cancer problems, Wales also has a unique set of attributes which make it an ideal place for cancer research. We are a relatively small country, both in terms of our compact geography and small population size. Our population is relatively static; people who live in Wales, on the whole, stay in Wales, making it easier to assess changes in the population’s health. Our small size allows greater collaboration between our academic institutions and NHS Wales, without the hurdle of large distances. In addition, Wales is blessed with some wonderfully talented cancer researchers, in our hospitals and universities. The Welsh Government works with both our own fantastic ‘local’ charities, such as Cancer Research Wales and Tenovus Cancer Care, and international cancer research funders, such as Cancer Research UK, to support internationally-recognised resources such as the Wales Cancer Bank, the Wales Gene Park, the Cardiff Experimental Cancer Medicine Centre, and the Life Sciences Research Network Wales.

The Wales Cancer Research Centre is funded by the Welsh Government (£4.5million, 2015-18) and is a vital part of NHS Wales’ research funding. Working within Health and Care Research Wales’ infrastructure, we bring academic and clinical researchers together, for the benefit of cancer patients, their families and the people of Wales and beyond. We employ over 40 members of staff at all levels of research, including nurses, doctors, laboratory researchers and pharmacists. Our dedicated staff conduct research at every stage, from understanding the cellular and molecular basis of cancer to interventions that improve the health and wellbeing of individual cancer patients. In order to deliver on all aspects of this spectrum of research activity, we have established four themes under which our work is conducted:

- **Pre-clinical:** We develop new treatments in a laboratory setting with a focus on advances in genetics, immunology, stem cell and drug development research.
- **Translational:** We take discoveries from the lab to the patients who can benefit, and enable scientists, using samples from patients, to understand cancer better.
- **Clinical:** We aim to increase recruitment to clinical trials, across a range of important cancer types.
- **Community:** We focus on three areas of research for the benefit of the public. These areas are: palliative and supportive care; screening, prevention and early diagnosis; health informatics.

Since our inception, in April 2015, the WCRC has been bringing hope to cancer patients across Wales, and beyond. We are now nearing the end of our second year and have seen fantastic outputs from the Centre. Our staff have secured 81 grants during our first 18 months of operation, totaling £8.6m. With these funds, an additional 44 whole time equivalent research posts have been brought into Wales.

The WCRC has a strong emphasis on collaboration. We are bringing together several areas of existing strength in Wales to advance research, build knowledge and bring inward investment into Wales by working with cancer charities, research councils, the pharmaceutical industry and other Health and Care Research Wales funded centres. An excellent example of this collaboration can be seen through our recent work developing a new breast cancer drug in Wales that will be given first to Welsh patients, within NHS Wales. We also work with the public through holding engagement events, involving the public in research and raising awareness of how research is conducted.

WCRC has an all-Wales brief, with a vision to work with cancer patients and other partners to deliver cancer research excellence in Wales. Our research partners include Bangor University, Cardiff University, Swansea University, the Velindre Cancer Centre and three of the University Health Boards within NHS Wales (Abertawe Bro Morgannwg, Aneurin Bevan, and Cardiff and Vale). At every stage of our work, we involve public and patient representatives. This is based on our strong conviction that ordinary people should not just be the subjects of research, but be active participants in our research, working with us to plan, conduct and present our work. We are particularly proud of the Patient and Public Involvement and Engagement team we have assembled, which includes members of the public who are already doing great work in helping us move our cancer research forward.

Based on this success, we will be applying to Health and Care Research Wales for a 2-year extension to our funding (2018-2020). This will allow our Centre to continue to perform and support cancer research of the highest quality, taking in the whole picture, from early laboratory studies, through top-quality clinical trials to improving the quality of life for people living with cancer in Wales. Fighting cancer is a huge challenge, but we are already curing more cancers than ever before. There is still much to be done. We believe that, one day soon, by working together, within Wales and internationally, cancers will meet their match. We hope that the work of the Wales Cancer Research Centre will now bring that victory one step closer.

To find out more about our work or, better still, to get involved, visit our website at www.walescancerresearchcentre.com, send us an email via WCRC@Cardiff.ac.uk, or call us on 02921 845852.

